# Contact Lens Health Week — August 20–24, 2018

**DOI:** 10.15585/mmwr.mm6732a1

**Published:** 2018-08-17

## Abstract

August 20–24, 2018, marks the fifth annual Contact Lens Health Week. In collaboration with partners from clinical, public health, industry, and regulatory sectors, CDC is promoting healthy contact lens wear and care practices to reduce the risk for eye infections among the approximately 45 million persons in the United States who wear contact lenses. Research after outbreaks of rare but serious eye infections in the United States has indicated that these infections occur most often in contact lens wearers who do not take proper care of their contact lenses, indicating a need to promote safer wear and care (*1*,*2*).

A report in this issue of *MMWR* reviews cases of contact lens–related eye infections associated with sleeping in contact lenses. Other reported habits in addition to sleeping while wearing lenses were swimming while wearing lenses and not replacing lenses and storage cases as often as recommended. Some of the patients sought care in an emergency department where it is more costly to receive care, and some of the infections led to serious adverse health outcomes.

Contact lenses can pose an infection risk, especially if they are not worn and cared for properly. Practicing proper contact lens hygiene is important for keeping contact lens wearers’ eyes healthy.

Additional information on Contact Lens Health Week and the proper wear and care of contact lenses is available at https://www.cdc.gov/contactlenses.

CDC receives an annual contribution from the Contact Lens Institute to support CDC’s Healthy Contact Lens Program. The Contact Lens Institute had no involvement in the drafting or review of this report.
